# Surgical Management of Radicular Cyst With the Use of Advanced Platelet-Rich Fibrin and Bone Graft: A Case Report

**DOI:** 10.7759/cureus.60742

**Published:** 2024-05-21

**Authors:** Simran Kriplani, Shweta Sedani, Aditya Patel, Manoj Chandak, Unnati Shirbhate, Joyeeta Mahapatra, Akash Thakare

**Affiliations:** 1 Department of Conservative Dentistry and Endodontics, Sharad Pawar Dental College and Hospital, Datta Meghe Institute of Higher Education and Research, Wardha, IND; 2 Department of Periodontics, Sharad Pawar Dental College and Hospital, Datta Meghe Institute of Higher Education and Research, Wardha, IND

**Keywords:** infected radicular cyst, bone regeneration, endodontic treatment, advanced platelet-rich fibrin, platelet-rich fibrin

## Abstract

In addition to helping with wound healing, periapical surgery is performed to remove periapical disease. Concentrates of platelets have been applied extensively in endodontics and other fields of regenerative medicine. A periapical inflammatory lesion was found in a 35-year-old male patient who complained of pain in the maxillary anterior region and displayed slight edema in the same area. The lesion was treated with periapical surgery utilizing advanced platelet-rich fibrin (A-PRF). Mesenchymal stem cell processes of proliferation and differentiation can be induced by several types of platelet concentrates. Growth factors are released at the application site by platelet-rich fibrin (PRF) for a minimum of seven days. The activity of osteoblasts is stimulated by growth factors and secreted cytokines. Furthermore, the release of growth factors promotes fibroblast migration, which quickens tissue regeneration.

In addition to helping with wound healing, periapical surgery is performed to remove periapical disease. The synthesis of fibrin networks laden with platelets and growth factors is made possible by PRF, which is subsequently used to accelerate bone regeneration and, consequently, to improve bone formation. In this instance, the best possible bone regeneration and repair were accomplished. After 12 weeks, 24 weeks, and 36 weeks, the patient was brought back for follow-ups. He was found to be asymptomatic, and the radiograph showed considerable periapical healing with nearly enough bone production.

## Introduction

Anterior tooth trauma is one of the frequent unplanned incidents that results in discomfort, deformity, and mental health issues. The primary stay of nonsurgical care for periapical pathologies, or symptomatic diseases of the necrosed pulp, is root canal cleaning [1].

One common inflammatory odontogenic cystic lesion that arises from epithelial remains in the periodontal ligament is the radicular cyst. Its existence is frequently overlooked in clinical settings, but radiographic analysis can reveal its presence. Consequently, enucleation and marsupialization are frequently used in surgical treatment to eliminate these cysts. If traumatically injured teeth are left untreated, they may develop cyst-%like apical periodontitis [2].

For the management of such situations, treatment methods such as periapical surgery have been suggested. When traditional root canal therapy is ineffective for treating teeth with periapical lesions, surgical endodontics is a dependable alternative. More than 80% of instances have been reported to have had successful results. Modern surgical methods, magnifying glasses, microsurgery tools, ultrasonic retro-tips, and better root end-filling materials could all be contributing factors to this high success rate [3-5]. Apical granulation tissue, also known as periradicular bone resorption and the breakdown of the apical periodontal ligament, is the outcome of the reactions caused by microbial infections and host response events. Cysts develop from some of the apical granulomas. There is a reported range of 6% to 54% for the production of periapical cysts [6-8].

## Case presentation

**Figure 1:**
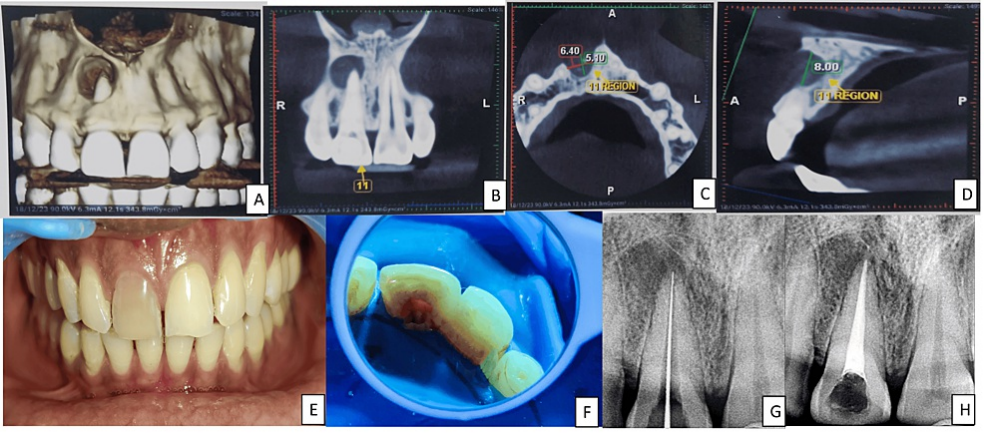
(A) CBCT with tooth number 11, (B) coronal section with tooth number 11 showing large periapical lesion, (C) axial section with tooth number 11 showing the extent of the lesion, (D) sagital section with tooth number 11 showing the lesion diameter of 8 mm, (E) intraoral picture showing discoloration with tooth number 11, (F) access opening with tooth number 11, (G) RVG of working length with tooth number 11, (H) RVG of obturation with tooth number 11 CBCT: Cone-beam computed tomography; RVG: radiovisiography

A 30-year-old male patient arrived at the Department of Endodontics and Conservative Dentistry. He reported having discolored anterior teeth in the upper left front region of his jaw, and five years before reporting, he had experienced damage from a car accident. Initially, after trauma, the patient was asymptomatic but then started experiencing pus discharge in the same region a year ago. A neural sensibility test was done with 12, 11, 21, and 22. The normal response was seen with 12, 21, 22, and 11, which showed no response. Heat tests using gutta-percha revealed the same results. The preoperative cone-beam computed tomography (CBCT) was advised, as shown in Figures 1A-1D, forming the final diagnosis of an infected radicular cyst with 11. The intraoral picture seen in Figure 1E depicts a discolored tooth, with 11 suggesting pulp necrosis. The nonsurgical treatment was planned earlier and informed consent was obtained, aiming at complete chemo-mechanical preparation and copious irrigation to disinfect the root canals.

Visit 1

Under proper isolation using OptraDam, access opening was done with 11, as seen in Figure 1F. Biomechanical preparation with 11.2 visits of calcium hydroxide dressing and two dressings of triple antibiotic paste (TAP) were given. There was no change observed in three months, so surgical treatment was opted for following the cystic enucleation and by apicectomy, retrograde mineral trioxide aggregate (MTA) filling, and administration of PRF and a bone graft. The working length of 11 was recorded as 21 mm. The canal was irrigated using 0.9% saline and 2% chlorhexidine. Calcium powder mixed with chlorhexidine was placed in the canal, followed by a temporary restoration.

Visit 2

The patient was recalled after 10 days. The calcium powder was removed thoroughly with 0.9% saline. Biomechanical preparation was performed using K files up to 70 no. 11 using the step-back technique as shown in Figure 1G. Irrigation was done with saline and chlorhexidine. Then, calcium powder mixed with chlorhexidine was placed, followed by a temporary restoration.

Visit 3

The patient was recalled after 10 days. Irrigation was done with saline to remove the calcium powder from the canal. The TAP was placed, followed by a temporary restoration. This was followed by two more visits, and then a master cone, gutta-percha 70 with tooth number 11, was selected and verified on radiovisiography. Obturation was done with System B backfill 11 using a bioceramic sealer, as seen in Figure 1H.

**Figure 2:**
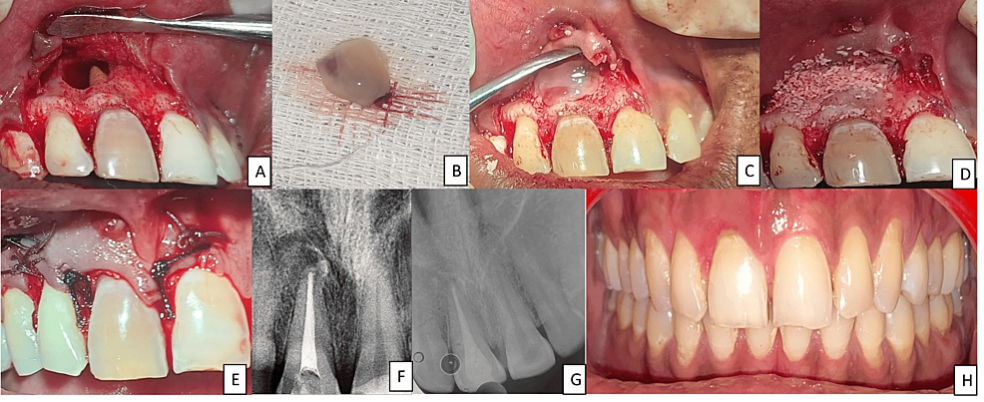
(A) The periapex of tooth number 11 showing the infected site with bony defect, (B) A-PRF prepared, (C) placement of A-PRF in the defect site, (D) bone graft placed in adjunct to A-PRF, (E) closure of the defect site with the help of sutures, (F) three-month follow-up IOPA, (G) six-month follow-up IOPA, (H) follow-up intraoral picture of nonvital bleaching with tooth number 11 A-PRF: Advanced platelet-rich fibrin; IOPA: intraoral periapical radiograph

**Figure 3:**
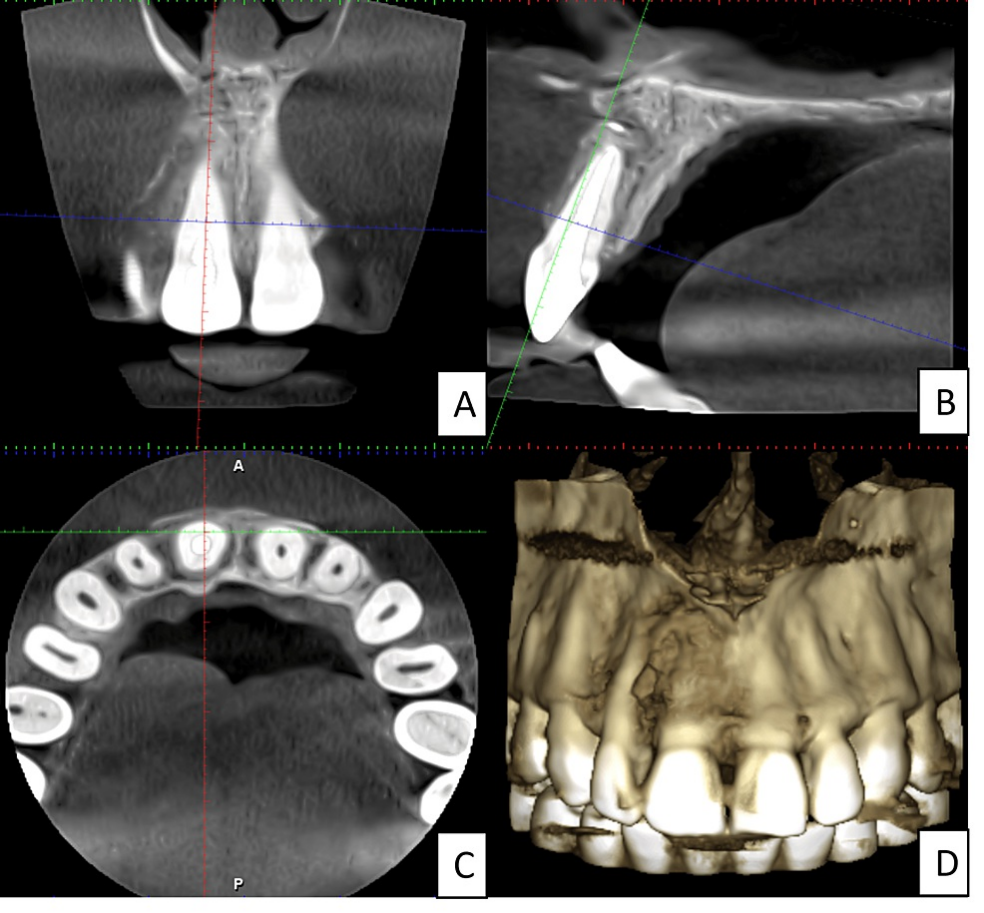
(A) One-year follow-up CBCT showing the coronal section with tooth number 11, (B) CBCT showing the sagittal section with tooth number 11, (C) CBCT showing the axial section with tooth number 11, (D) CBCT showing the skeletal view CBCT: Cone-beam computed tomography

The lesion was exposed, as depicted in Figure 2A. The A-PRF preparation procedure is a simple method created in France by Choukroun et al. [9]. Venous blood is extracted and placed in dry glass tubes, which are centrifuged at 3000 revolutions per minute (Process Protocols, Nice, France). Since no anticoagulants are used, immediate platelet activation and fibrin polymerization occur. Consequently, upon centrifugation, three distinct layers are formed: a base layer of red blood cells (RBCs), a top layer of acellular plasma, and a central clot of PRF. This PRF clot concentrates most of the platelets and leukocytes from the extracted blood into a strong fibrin matrix with a complicated three-dimensional architecture. The PRF clot is then inserted into the PRF Box (Process Ltd., Nice, France) grid and sealed with a lid and compressor. In about one minute, this method produces an affordable autologous fibrin membrane. Membranes with a constant thickness of 1 mm are produced by the PRF Box, as illustrated in Figure 2B. The serum exudate produced by the fibrin clots is retained by these membranes even after being hydrated for several hours. Proteins like vitronectin and fibronectin are abundant in this exudate. You can use the collected exudate in the bottom of the box to preserve autologous grafts, clean surgical sites, and hydrate graft materials. Alternatively, the clot can be pressed between two gauzes to remove extra fluid in order to generate a PRF membrane [10]. Another option is to feed the PRF clot into the PRF Box's cylinder, where it will be gradually compressed with a piston to form thick, 1-cm-diameter PRF "plugs." These stoppers are useful for safeguarding extraction locations. Surgical intervention was planned, and a local anesthetic (2% lignocaine) with adrenaline (1:1,00,000) was administered. A crevicular incision was given from the distal of 12 to the distal of 21. The releasing incision was given mesial to 12 and 21. A mucoperiosteal flap was elevated using a periosteal elevator. The tissue was removed and sent for histopathological examination. The curettage of the cystic cavity was performed using curettes. Hemostasis was achieved using vasoconstrictor-soaked cotton. Apicectomy was performed with 11 using a round bur on the micromotor with irrigation. Retrograde MTA filling was done, and A-PRF and bone grafts were placed as portrayed in Figures 2C-2D, respectively. The flap was closed, and Vicryl's simple, interrupted sutures were given, as shown in Figure 2E. RVG was taken after the entire procedure, as shown in Figure 2F. One day after surgery, swelling was noted, and there was no postoperative pain. Nonvital tooth bleaching was performed with tooth number 11, following walking bleach technique. Three visits were completed, and the desired tooth shade was achieved. Six months of follow-up: The swelling was completely resolved, and the patient did not experience any discomfort during the period. Intraoral periapical radiograph seen in Figure 2G. The intraoral image in Figure 2H depicts the result of the nonvital bleaching performed. One-year follow-up depicted enhanced healing with bone formation in the periapex of tooth number 11. CBCT evaluation, as seen in Figures 3A-3D, shows no periapical pathology with adequate bone healing.

## Discussion

Roughly half of all periapical lesions are radicular cysts, which are the most frequent type of cyst, making up 15% of all periapical lesions. With an incidence rate of 52.3%-70.7%, the periapical cyst is the most prevalent type of odontogenic cyst. It is followed by the dentigerous cyst (16.6%-21.3%) and the odontogenic keratocyst (5.4%-17.4%) [11].

Apical surgery, which has an 86%-92% success rate, is the main method available to treat a significant nonhealing periapical lesion when standard orthograde endodontic treatment fails [12]. The oldest known treatments for cystic lesions of the jaws are likely marsupialization and decompression, which were initially proposed by Partsch in German literature in the late 1800s. Decompression refers to any technique used to lower a cyst's internal pressure. In its truest sense, marsupialization refers to the transformation of the cyst into a pouch, implying the formation of a substantial, self-maintaining stoma. One technique for decompressing a cyst is marsupialization [13]. Complete removal of the cyst along with the lining is called enucleation.

Cystic enucleation allows for a cystic cavity to be covered by a mucoperiosteal flap, and the space fills with a blood clot, which will eventually organize and form normal bone. Small cysts can be enucleated intraorally; large cysts are to be enucleated extraorally. Enucleation with primary closure and reconstruction/bone grafting are desired for a successful treatment outcome [14].

A-PRF is affordable and simple to make from the patient's blood and may even be a highly helpful component for speeding up the healing of wounds. All the components beneficial for immunity and healing detected in a blood sample are present in the A-PRF. It is a concentration of platelets and the immune system building up on a single fibrin membrane. This novel biomaterial mimics an autologous cicatricial matrix while not being either fibrin glue or a typical platelet concentration. The efficacy of A-PRF treatment is rapidly determined by the time it takes to draw blood and put it into the centrifuge. Concentrated fibrinogen must be obtained in the center and upper parts of the tube by centrifugation for a short period, as blood coagulates when it comes into contact with anticoagulant-free tubes [14]. Prompt manipulation is necessary and the only practical way to produce a therapeutically useful A-PRF. Longer centrifuging and blood collection times will render the process unsuccessful. It will cause the fibrin that was collected in the tube to diffusely polymerize, and very little consistent fibrinogen will be produced [15]. In PRF platelets, growth factors constitute the predominant material. Transforming growth factor (TGF)-1 and 2, insulin-like growth factor (IGF), platelet-derived growth factor (PDGF), vascular epidermal growth factor (EGF), fibroblast growth factors, and EGF are a few of the several growth factors contained in PRF. These elements may possibly be able to regulate cell division in addition to being assumed to be crucial for bone metabolism. PDGF activates collagenase, which fortifies the repaired tissue. Fibroblast activator TGF promotes procollagen synthesis, which leads to the deposition of collagen in the wound. Furthermore, PRF promotes healing by lowering the local inflammatory response [16]. The process of bone regeneration in the afflicted area will be accelerated by implanting a bone graft following periapical surgery and cyst excision. As it is gradually absorbed to allow replacement by new bone tissue, the bone transplant serves as a base for the development of new bone. In addition, bone grafts serve as osteoconductive materials by stabilizing blood clots, promoting bone regeneration, and facilitating the migration of osteoprogenitor cells via a scaffold [17]. Therefore, while doing periapical operations, PRF may be one of the best biograft materials for filling bone defects.

## Conclusions

Applying the A-PRF membrane to surgically treat radicular cysts causes a gradual and sustainable release of important growth factors, stimulating the surrounding tissue for a considerable amount of time while the cyst is remodeling. When used as a healing biomaterial after periapical surgery, A-PRF has proven to be effective in the clinic. Because A-PRF offers patients a more affordable choice during surgery, clinicians should be aware of its advantages and employ it when performing procedures.
